# Reduced circulating dendritic cells in acute *Plasmodium knowlesi* and *Plasmodium falciparum* malaria despite elevated plasma Flt3 ligand levels

**DOI:** 10.1186/s12936-021-03642-0

**Published:** 2021-02-16

**Authors:** Jessica R. Loughland, Tonia Woodberry, Damian Oyong, Kim A. Piera, Fiona H. Amante, Bridget E. Barber, Matthew J. Grigg, Timothy William, Christian R. Engwerda, Nicholas M. Anstey, James S. McCarthy, Michelle J. Boyle, Gabriela Minigo

**Affiliations:** 1 Menzies School of Health Research and Charles Darwin University, Darwin, Australia; 2 QIMR Berghofer Medical Research Institute, Brisbane, Australia; 3 Gleneagles Hospital, Kota Kinabalu, Sabah Malaysia; 4 Infectious Diseases Society Kota Kinabalu Sabah-Menzies School of Research Clinical Research Unit, Kota Kinabalu, Sabah Malaysia; 5 Queen Elizabeth Hospital-Clinical Research Centre, Ministry of Health, Kota Kinabalu, Malaysia; 6 Royal Darwin Hospital, Darwin, Australia; 7 College of Health and Human Sciences, Charles Darwin University, Darwin, Australia

**Keywords:** IBSM, *Plasmodium falciparum*, *Plasmodium knowlesi*, Dendritic cells, CD141, BDCA3, Flt3 ligand, BDCA1, Plasmacytoid, CHMI

## Abstract

**Background:**

*Plasmodium falciparum* malaria increases plasma levels of the cytokine Fms-like tyrosine kinase 3 ligand (Flt3L), a haematopoietic factor associated with dendritic cell (DC) expansion. It is unknown if the zoonotic parasite *Plasmodium knowlesi* impacts Flt3L or DC in human malaria. This study investigated circulating DC and Flt3L associations in adult malaria and in submicroscopic experimental infection.

**Methods:**

Plasma Flt3L concentration and blood CD141^+^ DC, CD1c^+^ DC and plasmacytoid DC (pDC) numbers were assessed in (i) volunteers experimentally infected with *P. falciparum* and in Malaysian patients with uncomplicated (ii) *P. falciparum* or (iii) *P. knowlesi* malaria.

**Results:**

*Plasmodium knowlesi* caused a decline in all circulating DC subsets in adults with malaria. Plasma Flt3L was elevated in acute *P. falciparum* and *P. knowlesi* malaria with no increase in a subclinical experimental infection. Circulating CD141^+^ DCs, CD1c^+^ DCs and pDCs declined in all adults tested, for the first time extending the finding of DC subset decline in acute malaria to the zoonotic parasite *P. knowlesi*.

**Conclusions:**

In adults, submicroscopic *Plasmodium* infection causes no change in plasma Flt3L but does reduce circulating DCs. Plasma Flt3L concentrations increase in acute malaria, yet this increase is insufficient to restore or expand circulating CD141^+^ DCs, CD1c^+^ DCs or pDCs. These data imply that haematopoietic factors, yet to be identified and not Flt3L, involved in the sensing/maintenance of circulating DC are impacted by malaria and a submicroscopic infection. The zoonotic *P. knowlesi* is similar to other *Plasmodium* spp in compromising DC in adult malaria.

## Background

Human blood dendritic cells (DCs) are a heterogeneous population of antigen presenting cells, comprising several subsets with distinct phenotypes and functions [1]. Peripheral blood DCs can be divided into myeloid DCs (mDCs or classical DCs) and plasmacytoid DCs (pDCs), and are identified by their surface expression of CD11c and CD123, respectively [2]. There are three phenotypically distinct subpopulations of mDCs based on expression of CD1c (BDCA1^+^, DC2), CD16^+^ (FcγRIII) and CD141^+^ (BDCA3^+^, DC1) [1, 3]. DCs are key activators of the adaptive immune response to pathogens [4], including the malaria causing parasite *Plasmodium* [5]. Previous studies have shown that during experimental blood-stage *Plasmodium* infection [6–10], and in adults with uncomplicated *Plasmodium falciparum* and *Plasmodium vivax* malaria [11–13] numbers of all DC subsets are reduced in the periphery. The effect of the zoonotic parasite *Plasmodium knowlesi* on circulating DC subsets in human malaria is yet to be determined.

DC homeostasis is in part regulated by the haematopoietic cytokine FMS-like tyrosine kinase 3 ligand (Flt3L) [14–16]. A role for Flt3L in DC development was uncovered when mice and humans were administered Flt3L and DC numbers subsequently expanded [14, 17]. Further, bone marrow progenitors treated with FLT3L, preferentially support the development of mature DCs in vitro [18]. In murine malaria, Flt3L preferentially stimulates the expansion of CD8α^+^ CD103^+^ DCs which activate CD8^+^ T cells [19]. In humanized mice, Flt3L is associated with CD141^+^ DC expansion, and the maintenance and expansion of other DC subsets including CD1c^+^ DCs and pDC [15].

In children with severe *P. falciparum* malaria, *Plasmodium*-induced Flt3L is reported to selectively stimulate the expansion of blood CD141^+^ DCs, but not other DC subsets [19]. Increased Flt3L is observed in adults with uncomplicated *P. falciparum* malaria [20], yet associations with DCs have not been investigated in adults. Here, plasma Flt3L and circulating DC numbers were quantified in adults experimentally infected with *P. falciparum,* and adults with clinical *P. falciparum* or *P. knowlesi* malaria to better understand the impact of a *Plasmodium* infection on circulating DC numbers and their association with plasma Flt3L levels.

## Methods

### Ethics statement

Written and informed consent was obtained from all participants. The clinical malaria study in Malaysia was approved by the ethics committees of Menzies School of Health Research (HREC 10/1431) and the Malaysian Ministry of Health (NMRR 10-754-6684). Volunteer infection studies were approved by the Human Research Ethics Committees of QIMR Berghofer Medical Research Institute (P1479) and Menzies School of Health Research (HREC 10/1431).

### Cohorts

#### Clinical malaria

DC number were assessed in cryopreserved PBMCs and Flt3L in plasma collected from patients with uncomplicated *P. falciparum* (n = 11) or *P. knowlesi* malaria (n = 14) participating in a pathophysiology study at Queen Elizabeth and Kudat District Hospitals in Sabah, Malaysia [21, 22]. Post treatment PBMC and plasma were collected 28 days after anti-malarial treatment (Table 1). PBMC and plasma samples from PCR *Plasmodium*-negative adult visitors on the infectious disease ward were evaluated as controls (n = 15).

**Table 1:** Acute malaria patients and induced blood-stage malaria (IBSM) cohort information

	*P. falciparum*	*P. knowlesi*	IBSM	Healthy controls
Number	11	14	33	15
Median age in years [IQR]	42 [23–53]	42 [23–58]	24 [22–27]	44 [20–53]
Female, number (%)	6 (55)	3 (21)	17 (51)	8 (53)
Median parasite density^a^ (parasites/µl) [IQR]	15,503 [3264–36,977]	2158 [644–14,800]	5.3 [1.3–11.1]	NA

No significant difference in parasite density or age between *P. falciparum* and *P. knowlesi* patients

*IQR* interquartile range, *IBSM* induced blood-stage malaria

^a^Determined by PCR for IBSM and by microscopy for all other groups

#### Induced blood-stage malaria (IBSM) studies

For participants in experimental infection studies (n = 33), inoculum preparation, recruitment, infection and monitoring was performed as described previously [6, 23]. For assessment of CD141^+^ DCs, anticoagulated blood was collected prior to infection, 24 h prior to treatment, at the time of treatment and 24 h post treatment. Anti-malarial drugs were administered when volunteers reached a pre-determined parasitaemia threshold, median parasitaemia 5284 [IQR 1349–11,058] parasites/mL (day 7 or 8). Plasma samples were collected from volunteers before infection and at peak infection, plasma was cryopreserved within 30 min of blood collection. For CD141^+^ DCs characterization and function fresh whole blood was processed within 2 h of collection. Volunteers were recruited from clinical trials registered with US NIH ClinicalTrails.gov (ACTRN12611001203943, registered November 23, 2011; ACTRN12612000323820, registered March 21, 2012; ACTRN12612000814875, registered August 3, 2012; ACTRN12613000565741, registered May 17, 2013; ACTRN12613001040752, registered September 18, 2013; NCT02281344, registered October 3, 2014).

### Flt3L detection by ELISA

Plasma Flt3L levels were detected using human Flt-3 Ligand Quantikine ELISA kit (DFK00, R&D Systems), as per manufacturer’s instructions. Heparin plasma samples for each individual participant were tested in duplicate. Absorbance was measured at 450 nm and plasma Flt3L concentration was calculated using standards provided in the kit (minimum sensitivity less than 7 pg/mL). Mean concentration of duplicates tested is reported.

### DC subset enumeration

200 µL of fresh whole blood or 3 million PBMCs were stained at RT with surface antibodies, CD3 (HIT3a), CD14 (HCD14), CD19 (HIB19), CD56 (HCD56), HLA-DR (L243), CD11c (B-Ly6), CD123 (6H6), CD303 (201A), CD1c (L161), CD141 (M80), all antibodies were purchased from BD biosciences or Biolegend. For whole blood, RBCs were lysed with FACS lysing solution (BD) and cells fixed with 1% (w/v) paraformaldehyde in phosphate-buffered saline. Absolute numbers of DCs were determined by adding automated lymphocyte and monocyte counts (10^9^ cells/L), dividing the sum by 100, multiplying the percentage of DCs, and multiplying the product by 1000 to give the cell count/µL.

FACS data was acquired using a FACSCanto™ II (BD, USA) or Gallios™ (Beckman Coulter, USA) and data analysed using Kaluza^®^ 1.3 (Beckman Coulter, USA) and Flowjo V10.6 (BD, USA).

### Statistics

Statistical analyses used GraphPad Prism 6 (Graphpad Software Inc., USA). To compare within patient changes, the Wilcoxon matched-pairs sign rank test was used and the Mann–Whitney test was used to compare between patient changes (healthy controls *versus* malaria patients). Tests were two-tailed and considered significant with *p*-values < 0.05.

## Results

### CD141^+^ numbers significantly decline during primary subpatent *P. falciparum* infection without detectable changes in plasma FLT3L

**Fig. 1:**
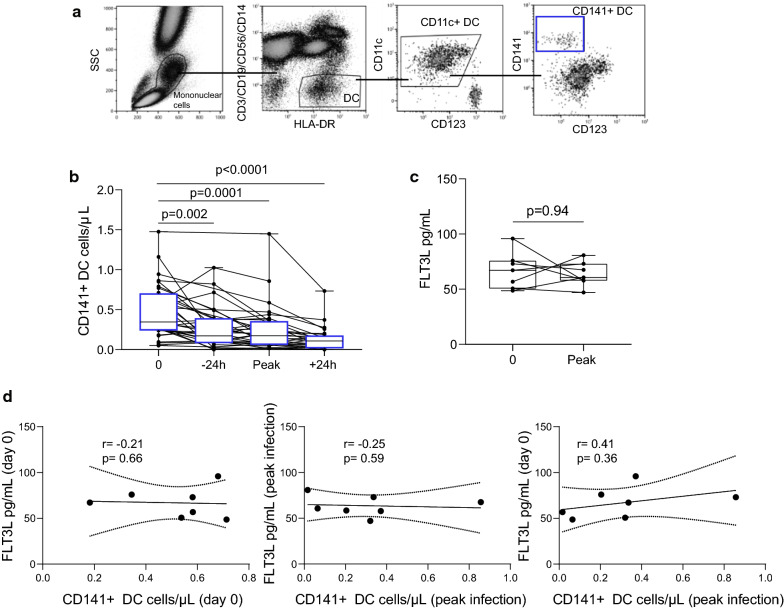
CD141^+^ DCs decline in malaria-naive volunteers experimentally infected with *P. falciparum*. **a** CD141^+^ DC gating. CD141^+^ DCs were identified as negative for lineage markers, HLA-DR^+^, CD11c^+^ and CD141^+^. **b** The absolute number of circulating CD141^+^ DCs in malaria naive volunteers experimentally infected with *P. falciparum* (d0; n = 33, − 24 h to peak; n = 26, peak; n = 33, + 24 h to peak n = 26). **c** Plasma Flt3L levels in malaria-naive volunteers experimentally infected with *P. falciparum* before infection (day 0) and at day predefined threshold for treatment reached (peak), n = 7. **d** Correlation of CD141^+^ DC number and Flt3L concentration on day 0 (left plot), correlation of CD141^+^ DC number and Flt3L concentration at peak infection (middle plot) and correlation of CD141^+^ DC number at peak infection and Flt3L concentration at day 0 (right plot) in volunteer infection studies. Box plots show the 10–90th percentile, median and interquartile range for data from all participants. The Wilcoxon matched-paired sign rank test was used to compare matched data. Spearman correlation was used for linear regression tests. Tests were two-tailed and considered significant if p-values < 0.05

CD1c^+^ DC and pDC numbers decline during experimental *P. falciparum* infection [6, 7]. Here, CD141^+^ DCs were measured in fresh whole blood collected from volunteers participating in experimental infection studies (Fig. 1a). Following primary experimental *P. falciparum* blood-stage infection, CD141^+^ DC numbers significantly decreased by day 6/7 (24 h before reaching the predefined threshold “peak” for treatment), and remained decreased for at least 24 h post treatment (Fig. 1b).

Plasma Flt3L was measured before infection (day 0) and at peak-infection. There was no change in plasma Flt3L levels (Fig. 1c). There was no correlation between plasma Flt3L and CD141^+^ DCs before infection, at treatment nor between pre-infection and the treatment time point (Fig. 1d). Similarly, there was no correlation between plasma Flt3L and other pDC or CD1c^+^ DC subsets analysed previously [6, 7] (Additional file 1: Fig. S1A, 1B).

### DC subsets decline in adults with acute uncomplicated *P. falciparum* or *P. knowlesi* malaria

**Fig. 2:**
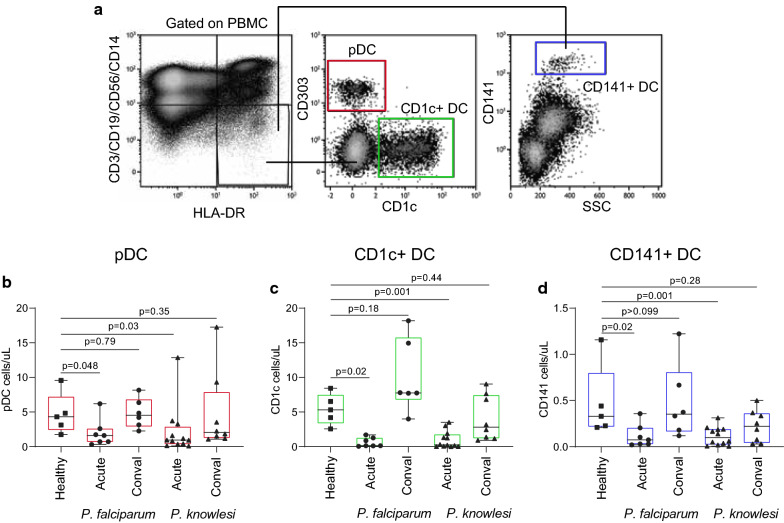
DC subsets decline in adults with acute clinical malaria. **a** DC subsets were identified as negative for lineage markers and HLA-DR^+^. Specifically, pDC were identified by CD303^+^ expression; CD1c^+^ DC were identified by CD1c^+^ (BDCA1) expression and CD141^+^ by CD141^+^ (BDCA3) expression. The absolute number of circulating **b** pDC (red), **c** CD1c^+^ DC (green) and **d** CD141^+^ DC (blue) in clinical malaria patients with *P. falciparum* (acute n = 7, conval = 6, circles) or *P. knowlesi* (acute n = 12, conval = 8, triangles) compared to local healthy controls (n = 5, healthy, squares). Box plots show the 10–90th percentile, median and interquartile range for data from all participants. The Mann–Whitney T-test was used to compare unpaired data (healthy vs acute and healthy vs conval). Tests were two-tailed and considered significant if p-values < 0.05

DC subset numbers in cryopreserved PBMCs were measured from patients with acute falciparum or knowlesi malaria as well as in-country uninfected healthy controls (Fig. 2a). During clinical malaria, plasmacytoid DCs (pDCs), CD1c^+^ DCs (BDCA1, DC2) and CD141^+^ DCs (BDCA3, DC1) numbers significantly decreased in both *P. falciparum* and *P. knowlesi* infection when compared to healthy uninfected controls (Fig. 2b–d). At convalescence (28 days post treatment), patient DC subset numbers recovered to comparable levels to healthy uninfected controls (Fig. 2b–d).

### Plasma Flt3L increased in adults with uncomplicated falciparum or knowlesi malaria

**Fig. 3:**
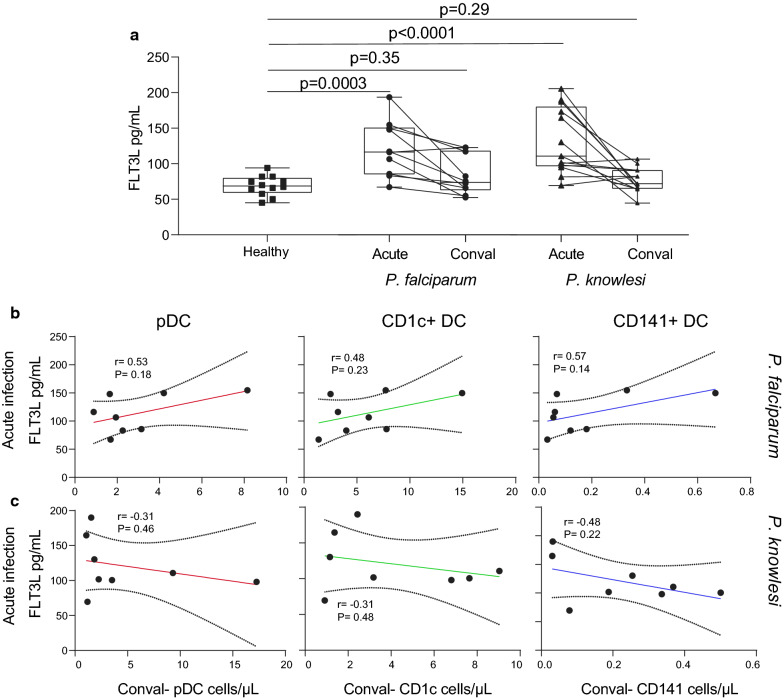
Increased plasma Flt3L in adults with acute *P. falciparum* or *P. knowlesi* malaria. **a** Plasma Flt3L levels in clinical malaria patients with either *P. falciparum*, (n = 10, circles) or *P. knowlesi* (n = 13, triangles) malaria (acute) compared to convalescence (conval) or compared to local uninfected healthy controls (n = 12, healthy, squares). Correlation of DC subset number at convalescence (conval) and Flt3L concentration at acute infection for pDC (left plots), CD1c^+^ DC (middle plots) and CD141^+^ DC (right plots) in **b*** P. falciparum* and **c** *P. knowlesi* clinical malaria. Box plots show the 10–90th percentile, median and interquartile range for data from all participants. The Wilcoxon matched-pairs sign rank test was used to compare matched data and Mann–Whitney T-test was used to compare unpaired data (healthy vs acute) and Spearman’s rank correlation was used for correlations. Tests were two-tailed and considered significant if p-values < 0.05

Plasma Flt3L was measured in adult patients with acute falciparum or knowlesi malaria and healthy uninfected controls. Plasma Flt3L was significantly increased in adults with falciparum (p = 0.0003) or knowlesi (p < 0.0001) malaria, compared to healthy uninfected local controls (Fig. 3a). At convalescence (28 days post treatment), patient Flt3L levels were comparable to healthy uninfected controls (Fig. 3a). There was no significant correlation between plasma Flt3L and parasitaemia in patients with falciparum or knowlesi malaria (Additional file 1: Fig. S2). There was no significant correlation between plasma Flt3L levels during acute infection and DC subset numbers at convalescence (Fig. 3b, c).

## Discussion

The relationship between DCs and Flt3L during malaria is incompletely understood. Here we show that similar to CD1c^+^ DCs and pDC subsets, circulating CD141^+^ DCs decline in numbers during experimental *P. falciparum* infection. Despite this decline, there was no detectable perturbation of plasma Flt3L in a first sub-microscopic infection in adults. Furthermore, the previously reported loss of pDC, CD1c^+^ DC and CD141^+^ DC subsets was confirmed in adults with acute falciparum malaria; and for the first time a significant decline in pDC, CD1c^+^ DC and CD141^+^ DC subsets in acute uncomplicated knowlesi malaria is reported. Previous reports show that plasma Flt3L is elevated in falciparum malaria, and here, for the first time elevated plasma Flt3L in patients with knowlesi malaria is reported, suggesting a pan *Plasmodium* effect of increased Flt3L in malaria. However, there was no association between plasma Flt3L and CD141^+^ DCs, in either adults with uncomplicated malaria or submicroscopic infection. These data may imply that other haematopoetic factors such as GM-CSF, M-CSF and/or IL-4 may be involved individually or in combination with Flt3L for the maintenance of circulating DC [24] and highlight the complicated relationship between DC subsets and Flt3L during malaria.

Early dysfunction of DCs during experimental malaria has been reported previously [6, 10]. Here, CD141^+^ DCs are also lost from peripheral circulation during experimental *P. falciparum* infection. Loss of CD141^+^ DCs during experimental *P. vivax* infection in healthy adult volunteers [25], in clinical *P. vivax* [12, 13] and now also clinical knowlesi malaria suggests a clear pan *Plasmodium* effect on CD141^+^ DCs in adults with subpatent or clinical malaria. Indeed, all DC subsets are reduced in both falciparum and knowlesi malaria. Changes in peripheral DC numbers during malaria may be explained by DC migration [26], DC apoptosis [13] or the failure to re-populate DCs from the bone marrow [27]. Increased expression of apoptotic markers annexin V [10] and caspase-3 [6] on DC subsets has been shown, suggesting, early apoptosis may contribute to premature loss of DCs. Future studies are required to establish whether the marked loss of CD141^+^ DCs from the circulation during *Plasmodium* infection is due to migration to organs such as the spleen and to identify haematopoietic factors that may be impaired by *Plasmodium* infection.

The plasma Flt3L increase observed in acute, uncomplicated falciparum or knowlesi malaria, are consistent with previous studies that found plasma Flt3L levels to be increased in clinical disease [19, 20]. In children, Flt3L levels are significantly higher in severe malaria patients compared to uncomplicated malaria patients [19], however, Flt3L concentrations reported in uncomplicated malaria in children are lower than those recorded in adults in this study and by others [20]. In adults with uncomplicated malaria, higher Flt3L levels are positively correlated with parasitaemia [20] yet, here there was no correlation with parasitaemia during clinical malaria. A parasite density threshold may trigger Flt3L release, via sensing of uric acid crystal accumulation by mast cells as demonstrated in *Plasmodium chabaudi* infection [19, 30]. In mice, release of Flt3L by mast cells was reported to drive the expansion of splenic CD8α^+^ DCs [19]. Only one study has assessed mast cell function in human malaria, with increased activation of mast cells in patients with severe malaria when compared to uncomplicated malaria patients [29]. In contrast to clinical malaria, no increase in Flt3L during experimental infection was observed. These data suggest a parasite threshold and/or symptomatic disease are associated with Flt3L elevation in malaria.

In the current study, no association between plasma Flt3L and CD141^+^ DC, CD1c^+^ DC or pDC peripheral cell numbers, in adults with clinical malaria, nor subpatent infection. Rather, despite increased Flt3L in clinical malaria, circulating CD141^+^ DCs and other DC subsets were significantly reduced. Further, Flt3L plasma levels and DC subset numbers were comparable between convalescent patients and healthy controls. However, the recovery of DC subsets was not correlated with Flt3L. These observations may be explained by the role of other haematopoietic cytokines including GM-CSF, M-CSF and IL-4 in DC development [30, 31]. In mice, the lack of Flt3L is compensated by increased production of M-CSF and IL-4, which results in continued expansion of DCs from progenitor cells [24]. Paradoxically, DC numbers significantly expand from progenitor cells when Flt3L receptor is deleted, suggesting multiple cytokines can induce DC development [24]. Future studies are required to assess M-CSF and other DC generating cytokines longitudinally in clinical malaria cohorts and the associations of these factors with DC subsets. The lack of an observed correlation between Flt3L and parasitaemia or DC subsets during clinical malaria could also be impacted by the limited longitudinal sampling within our cohorts and restricted sample numbers.

## Conclusions

In summary, similar to classical CD1c^+^ DCs (6), and pDCs (7) CD141^+^ DCs are reduced during experimental *P. falciparum* infection. Similarly, this study shows that pDC, CD1c^+^ DC and CD141^+^ DCs subsets are significantly reduced during clinical falciparum and for the first time, also in knowlesi malaria. These data suggest that Flt3L released in adults with uncomplicated malaria may not be sufficient to expand or restore circulating DC subsets during acute infection. Rather, multiple signals, not just Flt3L, may be required to expand CD141^+^ DCs and other DC subsets [30, 31].

## Supplementary Information

**Additional file** **1**: **Figure S1**. No significant correlation between plasma Flt3L and peripheral blood pDC or CD1c+ DC in induced blood-stage malaria. Correlations of DC subset numbers and Flt3L concentration on day 0 (left plot), correlation of DC subset numbers and Flt3L concentration at peak infection (middle plot) and correlation of DC subset numbers at peak infection and Flt3L concentration at day 0 (right plot) for **A. **pDC (red) and **B. **CD1c+ DCs (green) in volunteer infection studies. Spearman correlation was used and tests were two-tailed and considered significant if p-values <0.05. **Figure S2.** No significant correlation between plasma Flt3L and parasitemia in acute clinical malaria. Correlations between Flt3L concentration at acute infection and parasitemia (left plots), and correlations between Flt3L concentration at convalescence and parasitemia (right plots), for **A. ***P. falciparum* malaria and **B. ***P. knowlesi* malaria. Spearman correlation was used and tests were two-tailed and considered significant if p-values <0.05.

## Data Availability

All data generated or analysed during this study are included in this published article.

## References

[1] Villani A-C, Satija R, Reynolds G, Sarkizova S, Shekhar K, Fletcher J (2017). Single-cell RNA-seq reveals new types of human blood dendritic cells, monocytes, and progenitors. Science.

[2] Ju X, Clark G, Hart D (2010). Review of human DC subtypes. Methods Mol Biol.

[3] MacDonald KPA, Munster DJ, Clark GJ, Dzionek A, Schmitz J, Hart DNJ (2002). Characterization of human blood dendritic cell subsets. Blood.

[4] O’Keeffe M, Mok WH, Radford KJ (2015). Human dendritic cell subsets and function in health and disease. Cell Mol Life Sci.

[5] Yap XZ, Lundie RJ, Beeson J, O'Keeffe M (2019). Dendritic cell responses and function in malaria. Front Immunol.

[6] Loughland JR, Minigo G, Burel J, Tipping PE, Piera KA, Amante FH (2016). Profoundly reduced CD1c+ myeloid dendritic cell HLA-DR and CD86 expression and increased tumor necrosis factor production in experimental human blood-stage malaria infection. Infect Immun.

[7] Loughland JR, Minigo G, Sarovich DS, Field M, Tipping PE, Montes de Oca M (2017). Plasmacytoid dendritic cells appear inactive during sub-microscopic *Plasmodium falciparum* blood-stage infection, yet retain their ability to respond to TLR stimulation. Sci Rep.

[8] Loughland JR, Woodberry T, Boyle MJ, Tipping PE, Piera KA, Amante FH (2018). *Plasmodium falciparum* activates CD16^+^ dendritic cells to produce tumor necrosis factor and interleukin-10 in subpatent malaria. J Infect Dis.

[9] Teirlinck AC, Roestenberg M, Bijker EM, Hoffman SL, Sauerwein RW, Scholzen A (2015). *Plasmodium falciparum* infection of human volunteers activates monocytes and CD16+ dendritic cells and induces up-regulation of CD16 and CD1c expression. Infect Immun.

[10] Woodberry T, Minigo G, Piera KA, Amante FH, Pinzon-Charry A, Good MF (2012). Low-level *Plasmodium falciparum* blood-stage infection causes dendritic cell apoptosis and dysfunction in healthy volunteers. J Infect Dis.

[11] Jangpatarapongsa K, Chootong P, Sattabongkot J, Chotivanich K, Sirichaisinthop J, Tungpradabkul S (2008). *Plasmodium vivax* parasites alter the balance of myeloid and plasmacytoid dendritic cells and the induction of regulatory T cells. Eur J Immunol.

[12] Kho S, Marfurt J, Noviyanti R, Kusuma A, Piera KA, Burdam FH (2015). Preserved dendritic cell HLA-DR expression and reduced regulatory T cell activation in asymptomatic *Plasmodium falciparum* and *P. vivax* infection. Infect Immun.

[13] Pinzon-Charry A, Woodberry T, Kienzle V, McPhun V, Minigo G, Lampah DA (2013). Apoptosis and dysfunction of blood dendritic cells in patients with falciparum and vivax malaria. J Exp Med.

[14] Maraskovsky E, Daro E, Roux E, Teepe M, Maliszewski CR, Hoek J (2000). In vivo generation of human dendritic cell subsets by Flt3 ligand. Blood.

[15] Ding Y, Wilkinson A, Idris A, Fancke B, O’Keeffe M, Khalil D (2014). FLT3-ligand treatment of humanized mice results in the generation of large numbers of CD141+ and CD1c+ dendritic cells in vivo. J Immunol.

[16] Antonysamy MA, Thomson AW (2000). Flt3 ligand (FL) and its influence on immune reactivity. Cytokine.

[17] Maraskovsky E, Brasel K, Teepe M, Roux ER, Lyman SD, Shortman K (1996). Dramatic increase in the numbers of functionally mature dendritic cells in Flt3 ligand-treated mice: multiple dendritic cell subpopulations identified. J Exp Med.

[18] Naik SH, Proietto AI, Wilson NS, Dakic A, Schnorrer P, Fuchsberger M (2005). Cutting edge: generation of splenic CD8+ and CD8- dendritic cell equivalents in Fms-like tyrosine kinase 3 ligand bone marrow cultures. J Immunol.

[19] Guermonprez P, Helft J, Claser C, Deroubaix S, Karanje H, Gazumyan A (2013). Inflammatory Flt3l is essential to mobilize dendritic cells and for T cell responses during *Plasmodium* infection. Nat Med.

[20] Parigi SM, Czarnewski P, Das S, Steeg C, Brockmann L, Fernandez-Gaitero S (2018). Flt3 ligand expands bona fide innate lymphoid cell precursors in vivo. Sci Rep.

[21] Barber BE, William T, Grigg MJ, Menon J, Auburn S, Marfurt J (2013). A prospective comparative study of knowlesi, falciparum, and vivax malaria in Sabah, Malaysia: high proportion with severe disease from *Plasmodium knowlesi* and *Plasmodium vivax* but no mortality with early referral and artesunate therapy. Clin Infect Dis.

[22] Grigg MJ, William T, Barber BE, Rajahram GS, Menon J, Schimann E (2018). Age-related clinical spectrum of *Plasmodium knowlesi* malaria and predictors of severity. Clin Infect Dis.

[23] McCarthy J, Sekuloski S, Griffin PM, Elliott S, Douglas N, Peatey C (2011). A pilot randomised trial of induced blood-stage *Plasmodium falciparum* infections in healthy volunteers for testing efficacy of new antimalarial drugs. PLoS ONE.

[24] Durai V, Bagadia P, Briseño CG, Theisen DJ, Iwata A, Davidson JT (2018). Altered compensatory cytokine signaling underlies the discrepancy between Flt3–/–and Flt3l–/–mice. J Exp Med.

[25] Woodberry T, Loughland JR, Minigo G, Burel JG, Amante FH, Piera KA (2017). Early immune regulatory changes in a primary controlled human *Plasmodium vivax* infection: CD1c+ myeloid dendritic cell maturation arrest, induction of the kynurenine pathway, and regulatory t cell activation. Infect Immun.

[26] Pichyangkul S, Yongvanitchit K, Kum-arb U, Hemmi H, Akira S, Krieg AM (2004). Malaria blood stage parasites activate human plasmacytoid dendritic cells and murine dendritic cells through a Toll-like receptor 9-dependent pathway. J Immunol.

[27] Romani N, Gruner S, Brang D, Kämpgen E, Lenz A, Trockenbacher B (1994). Proliferating dendritic cell progenitors in human blood. J Exp Med.

[28] Furuta T, Imajo-Ohmi S, Fukuda H, Kano S, Miyake K, Watanabe N (2008). Mast cell-mediated immune responses through IgE antibody and Toll-like receptor 4 by malarial peroxiredoxin. Eur J Immunol.

[29] Wilainam P, Nintasen R, Viriyavejakul P (2015). Mast cell activation in the skin of *Plasmodium falciparum* malaria patients. Malar J.

[30] Mosca PJ, Hobeika AC, Colling K, Clay TM, Thomas EK, Caron D (2002). Multiple signals are required for maturation of human dendritic cells mobilized in vivo with Flt3 ligand. J Leukoc Biol.

[31] Jefford M, Schnurr M, Toy T, Masterman K-A, Shin A, Beecroft T (2003). Functional comparison of DCs generated in vivo with Flt3 ligand or in vitro from blood monocytes: differential regulation of function by specific classes of physiologic stimuli. Blood.

