# Supplementary material for: Reduced circulating dendritic cells in acute Plasmodium knowlesi and Plasmodium falciparum malaria despite elevated plasma Flt3 ligand levels
Source: Malar J. 2021 Feb 16;20:97. doi: 10.1186/s12936-021-03642-0 (PMC7888183; doi:10.1186/s12936-021-03642-0)

Plasmodium knowlesi and _P. falciparum_ malaria reduce circulating CD141 (BDCA-3), CD1c (BDCA-1) and plasmacytoid dendritic cells, and increase plasma Flt3 ligand.

Jessica R. Loughland[1,2*], Tonia Woodberry[2], Damian Oyong[2], Kim A. Piera[2], Fiona H. Amante[1], Bridget E. Barber[1,2,3], Matthew J. Grigg[2,3], Timothy William[3,4,5], Christian R. Engwerda[1], Nicholas M. Anstey[2,6], James S. McCarthy[1], Michelle J Boyle[1,2], Gabriela Minigo[2,7*]

[1]QIMR Berghofer Medical Research Institute, Brisbane, Australia; [2]Menzies School of Health Research and Charles Darwin University, Darwin, Australia; [3]Gleneagles Hospital, Kota Kinabalu, Sabah, Malaysia; [4]Infectious Diseases Society Kota Kinabalu Sabah-Menzies School of Research Clinical Research Unit, Kota Kinabalu, Sabah, Malaysia; [5]Queen Elizabeth Hospital-Clinical Research Centre, Ministry of Health, Malaysia' [6]Royal Darwin Hospital, Darwin, Australia; [7]College of Health and Human Sciences, Charles Darwin University, Darwin, Australia.

Supplementary Figure 1. No significant correlation between plasma Flt3L and peripheral blood pDC or CD1c$^+$ DC in induced blood-stage malaria. Correlations of DC subset numbers and Flt3L concentration on day 0 (left plot), correlation of DC subset numbers and Flt3L concentration at peak infection (middle plot) and correlation of DC subset numbers at peak infection and Flt3L concentration at day 0 (right plot) for **A.** pDC (red) and **B.** CD1c$^+$ DCs (green) in volunteer infection studies. Spearman correlation was used and tests were two-tailed and considered significant if p-values <0.05.

Supplementary Figure 2. No significant correlation between plasma Flt3L and parasitemia in acute clinical malaria. Correlations between Flt3L concentration at acute infection and parasitemia (left plots), and correlations between Flt3L concentration at convalescence and parasitemia (right plots), for **A.** *P. falciparum* malaria and **B.** *P. knowlesi* malaria. Spearman correlation was used and tests were two-tailed and considered significant if p-values <0.05.

Supplementary Figure 1

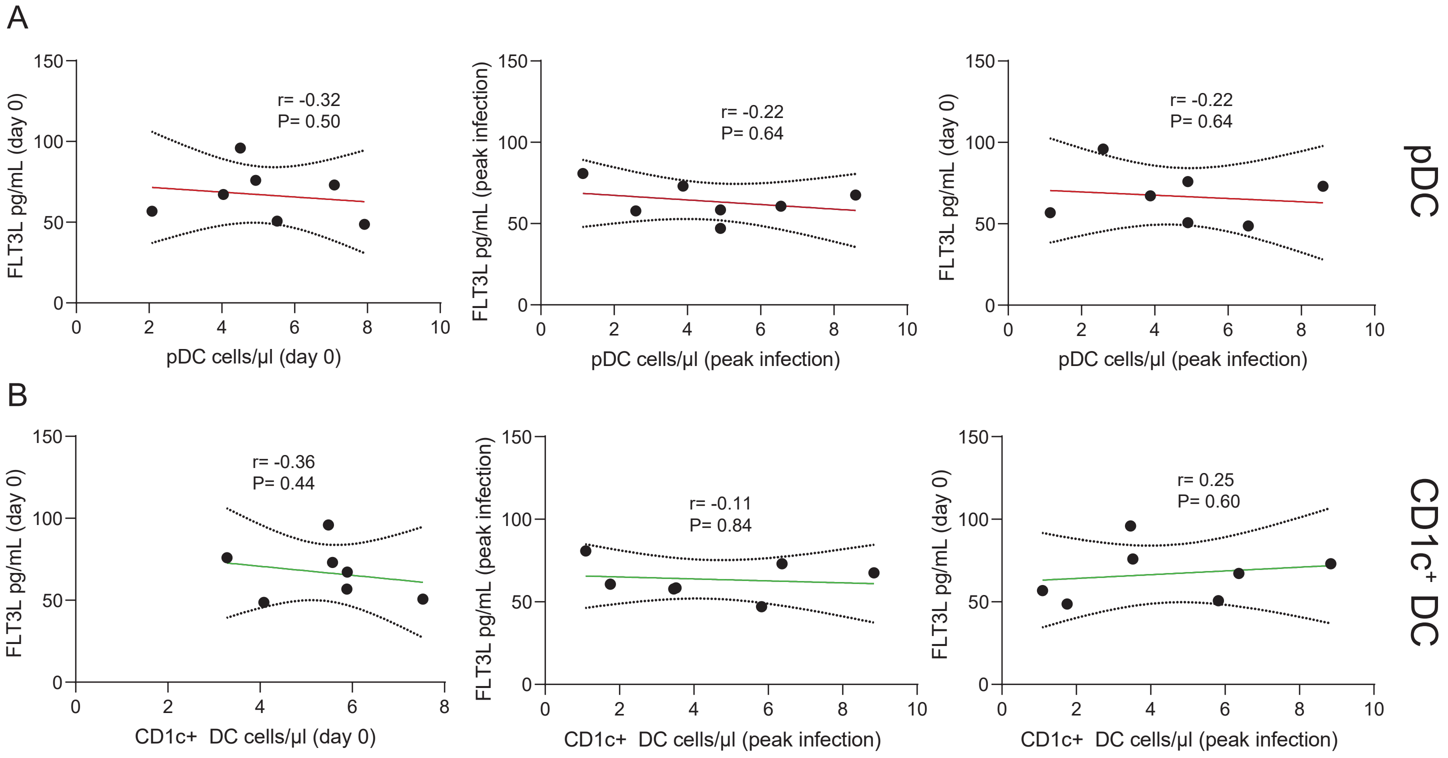

Supplementary Figure 2

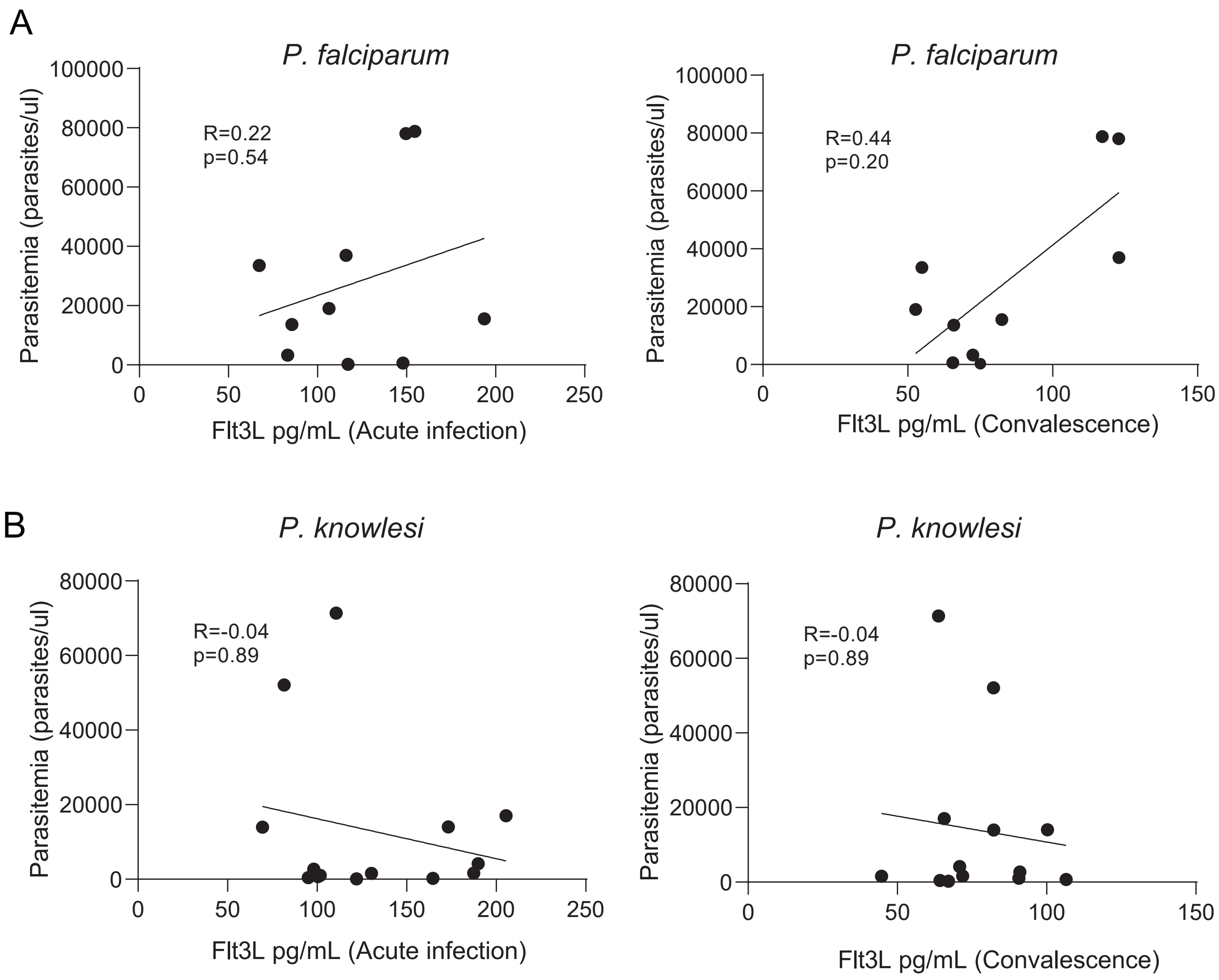

Supplement: Supplementary file 1 — Additional file 1: Figure S1. No significant correlation between plasma Flt3L and peripheral blood pDC or CD1c+ DC in induced blood-stage malaria. Correlations of DC subset numbers and Flt3L concentration on day 0 (left plot), correlation of DC subset numbers and Flt3L concentration at peak infection (middle plot) and correlation of DC subset numbers at peak infection and Flt3L concentration at day 0 (right plot) for A. pDC (red) and B. CD1c+ DCs (green) in volunteer infection studies. Spearman correlation was used and tests were two-tailed and considered significant if p-values <0.05. Figure S2. No significant correlation between plasma Flt3L and parasitemia in acute clinical malaria. Correlations between Flt3L concentration at acute infection and parasitemia (left plots), and correlations between Flt3L concentration at convalescence and parasitemia (right plots), for A. P. falciparum malaria and B. P. knowlesi malaria. Spearman correlation was used and tests were two-tailed and considered significant if p-values <0.05. [file 12936_2021_3642_MOESM1_ESM.pdf]
